# Exposure to Common Food Additive Carrageenan Alone Leads to Fasting Hyperglycemia and in Combination with High Fat Diet Exacerbates Glucose Intolerance and Hyperlipidemia without Effect on Weight

**DOI:** 10.1155/2015/513429

**Published:** 2015-03-25

**Authors:** Sumit Bhattacharyya, Leo Feferman, Terry Unterman, Joanne K. Tobacman

**Affiliations:** ^1^Department of Medicine, University of Illinois at Chicago, Chicago, IL 60612-4325, USA; ^2^Jesse Brown VA Medical Center, Chicago, IL 60612-3728, USA

## Abstract

*Aims*. Major aims were to determine whether exposure to the commonly used food additive carrageenan could induce fasting hyperglycemia and could increase the effects of a high fat diet on glucose intolerance and dyslipidemia.* Methods*. C57BL/6J mice were exposed to either carrageenan, high fat diet, or the combination of high fat diet and carrageenan, or untreated, for one year. Effects on fasting blood glucose, glucose tolerance, lipid parameters, weight, glycogen stores, and inflammation were compared.* Results*. Exposure to carrageenan led to glucose intolerance by six days and produced elevated fasting blood glucose by 23 weeks. Effects of carrageenan on glucose tolerance were more severe than from high fat alone. Carrageenan in combination with high fat produced earlier onset of fasting hyperglycemia and higher glucose levels in glucose tolerance tests and exacerbated dyslipidemia. In contrast to high fat, carrageenan did not lead to weight gain. In hyperinsulinemic, euglycemic clamp studies, the carrageenan-exposed mice had higher early glucose levels and lower glucose infusion rate and longer interval to achieve the steady-state.* Conclusions*. Carrageenan in the Western diet may contribute to the development of diabetes and the effects of high fat consumption. Carrageenan may be useful as a nonobese model of diabetes in the mouse.

## 1. Introduction

In a previous report, we identified exposure to the common food additive carrageenan as a cause of abnormal glucose tolerance and insulin resistance in C57BL/6J mice, due to impairment of insulin signaling by carrageenan-initiated inflammation [1]. In this report, we demonstrate the impact of exposure to a low concentration of carrageenan in the water supply on fasting blood sugar, hemoglobin A1c, serum lipids, hepatic glycogen stores, and weight gain and compare the effects to those of high fat diet and high fat diet with carrageenan. The interval to onset of glucose intolerance following exposure to carrageenan is also determined. These studies are highly relevant to the clinical development of diabetes, since carrageenan is consumed in comparable quantity in the typical Western diet to the level of exposure in the mice in these experiments.

In this report, we consider the combined effect of the common food additive carrageenan with the high fat diet, in comparison to high fat diet alone, recognizing that the high fat diet has been implicated in disease pathogenesis [2–5]. Carrageenan, which is composed of sulfated and unsulfated galactose residues, is a very commonly used additive in the Western diet, since addition of carrageenan to processed foods improves the texture of the processed foods. In latter decades of the twentieth century, the uses of carrageenan in manufactured foods accelerated, in order to improve texture with reduced fat content, as in low fat sandwich meats and dietetic supplements. Average daily consumption of carrageenan in the typical Western diet has been estimated to be 250 mg/day [6, 7]. This corresponds to about twice the quantity of carrageenan ingested by the experimental mice in this report (250 mg/60 kg/day = ~4.2 *μ*g/g/d in adult versus 10 *μ*g/mL × 5 mL/25 g/day = 2 *μ*g/g/d in mouse). The actual intake of carrageenan by adults may vary considerably based on food choices, and a recent industry review indicates intake ranging from 18 to 40 mg/kg/d [8].

In thousands of experiments conducted over several decades, exposure to carrageenan has been shown to predictably lead to inflammation [9]. Carrageenan-induced inflammation has been frequently used to test the effectiveness of anti-inflammatory medications. The mechanisms by which carrageenan causes inflammation include both activation of reactive oxygen species, leading to increased NF-*κ*B, and activation of innate immunity through the toll-like receptor TLR4-BCL10-mediated pathway, leading to increases in both RelA and RelB [10–12]. Carrageenan's activation of innate immunity derives from its fundamental biochemical structure, since it is composed of alternating galactose-*α*-1,3-galactose and galactose-*β*-1,4-galactose bonds [13]. The galactose-*α*-1,3-galactose epitope, also expressed as *α*-D-Gal-(1→3)-D-Gal or alpha-Gal, is foreign to humans and old world primates that lack the alpha-1,3-galactosyltransferase enzyme. Allergic IgE reactions to tick bites and to red meat have been associated with anti-Gal responses, as have anaphylactic reactions to cetuximab infusion [14–17]. 1%–3% of the United States population is reported to have anti-Gal antibodies, and anti-Gal antibodies are a major impediment to successful transplant to humans from most mammals [18–20]. Alpha-Gal antibodies were reported to be present on glycolipids and glycoproteins of nonprimate animals, including red meat of beef, pork, and lamb [16, 17]. Delayed anaphylaxis, angioedema, and urticaria have been reported after consumption of red meat in patients with IgE specific antibodies for galactose-*α*-1,3,-galactose [17], and both IgG and IgM anti-Gal antibodies are present in human serum [20, 21].

Prior studies demonstrated that exposure to carrageenan impaired insulin signaling in human HepG2 cells and in the liver of C57BL/6J mice, due to carrageenan-induced inflammation leading to increased phospho-Ser307-IRS1, an inhibitory mediator of insulin signaling that blocks phosphoinositide-3-kinase (PI3K) activity and the subsequent phosphorylation of AKT Ser473 in response to insulin stimulation [1, 22, 23]. Inhibition of carrageenan-induced inflammation reversed the carrageenan-induced increases in phospho-Ser307-IRS1 and in PI3K activity. Activation of inflammation through the TLR4 has been previously implicated in the etiology of diabetes [24–26], and carrageenan exposure leads to inflammation through interaction with the TLR4, as well as ROS [11, 27].

In prior work, evidence of glucose intolerance and insulin resistance was present after 18 days of carrageenan exposure. In this report, earlier impairment of glucose tolerance is presented, as well as aberrant responses to insulin and glucose infusion in hyperinsulinemic/euglycemic clamp studies. The effect of carrageenan intake on total cholesterol, triglycerides, HDL, and weight and the additive effect of carrageenan exposure to the impact of high fat diet alone on these parameters are detailed. Study data suggest that carrageenan exposure may be a useful model of diabetes in the nonobese mouse.

## 2. Methods

### 2.1. Carrageenan Ingestion and High Fat Diet in C57BL/6J Mice

Eight-week-old male C57BL/6J mice (*n* = 32 in groups of 8) were purchased (Jackson Laboratories, Bar Harbor, Maine, USA) and housed in the Veterinary Medicine Unit at the Jesse Brown VA Medical Center (JBVAMC, Chicago, IL, USA). All procedures were approved by the Animal Care Committee of the University of Illinois at Chicago and the JBVAMC. All mice were initially fed a standard diet and maintained with routine light-dark cycles. After acclimation to the environment, eight of the experimental animals received water with carrageenan (*λ*–*κ* high molecular weight carrageenan 10 mg/L; Sigma Chemical Co., St. Louis, MO, USA), eight were placed on a high fat diet (HFD; 58% fat (D12331), Research Diets, Inc., New Brunswick, NJ), eight received the high fat diet and carrageenan (HFD + carrageenan) in their water, and eight were untreated controls. Animals were weighed weekly. The experimental exposures continued for one year, at which time groups of mice were euthanized and tissues were harvested.

### 2.2. Glucose Determinations and Glucose Tolerance Test

Whole blood samples from a small tail incision were collected on glucose strips following a five-hour fast. Glucose levels were measured by glucometer (One Touch Ultra 2, LifeScan, Milpitas, CA, USA), as previously [1], and the average levels were compared among the groups. Glucose tolerance tests (GTT) were performed following overnight 15-hour fasts, with measurements at times 0, 15, 30, 60, and 90 minutes following dextrose injection (2 g/kg IP in filtered PBS). Mean glucose values from at least three mice from each group at each time point were compared.

### 2.3. Lipid Determinations

Serum lipid measurements, including high density lipoprotein (HDL; Sigma Chemical Co., St. Louis, MO; MAK045), total cholesterol, and triglycerides (Wako Diagnostic, Mountain View, CA), were performed by ELISA, using serum collected by orbital bleeding at ~one year of age.

### 2.4. Hyperglycemic-Euglycemic Metabolic Clamp Studies

Hyperglycemic-euglycemic clamp studies were performed at the Mouse Metabolic Phenotyping Center (MMPC) at Vanderbilt University [28]. Twenty-four 10-week-old male C57BL/6J mice were shipped to the MMPC from Jackson Laboratories. Bottles of sterile water with 10 mg/L of carrageenan (*λ*–*κ* high molecular weight carrageenan 10 mg/L; Sigma) were prepared in Dr. Tobacman's laboratory and shipped to the MMPC. Surgical procedures required for the metabolic studies were performed in control and carrageenan-exposed mice, as previously detailed [28]. The mice were studied on day 18 of carrageenan exposure after a 5-hour fast.

Procedures included insertion of a jugular venous catheter and a carotid artery catheter for infusion of glucose and insulin and for blood sampling [28]. Clamp studies were performed 48 hours following insertion of the catheters. Baseline blood sugar and insulin levels were drawn and infusions of insulin (Humulin Regular U100 at 4 mU/kg/min) were initiated at *t* = 0 and continued to *t* = 120. The rate of glucose infusion was adjusted using glucose measurements performed every five minutes throughout the experiment. 3-[^3^H]-D-Glucose was continuously infused throughout the study, and a bolus of ^14^C-2-deoxyglucose was infused at *t* = 120 minutes, at the conclusion of the study, to detect the rate of endogenous glucose production and the rate of glucose utilization in several tissues, including adipose tissue, heart, and brain. Experimental mice were transfused with blood from age- and gender-matched mice to maintain hemoglobin levels.

### 2.5. Hemoglobin A1c Determinations

Hemoglobin A1c was measured by ELISA (MyBioSource, San Diego, CA) in blood samples from the mice at ~1 year of age, following experimental carrageenan and/or high fat diet for ~44 weeks. Hemoglobin A1c is expressed as % of total hemoglobin.

### 2.6. Hepatic Glycogen Assay

Hepatic tissue was immediately frozen at the time mice were euthanized. Tissue homogenates were prepared and recommended assay procedures followed (MBL International, Woburn, MA). Glucoamylase hydrolyzed the glycogen to glucose, which was then oxidized and detectable at 570 nm. Glycogen detection range was from 0.0004 to 2 mg/mL.

### 2.7. Histochemistry for Detection of Glycogen Stores

Slides of hepatic tissue were prepared and stained using standard procedures for periodic acid Schiff staining [29] to detect glycogen stores. Photomicrographs were taken with a Motic imaging system (Carlsbad, CA), background color was changed to white by GIMP (GNU Image Manipulation Program), and the extent of cellular staining was compared among representative sections from the four groups of mice.

### 2.8. Measures of Colonic and Systemic Inflammation

Serum levels ofkeratinocyte-derived chemokine (KC), the mouse homolog of IL-8, were determined by ELISA (R&D, Minneapolis, MN) at ~one year of age. KC was expressed as pg/mL. Fecal calprotectin, a reliable measure of colonic inflammation [30], was determined by ELISA (Alpco Diagnostics, Salem, NH), following the recommended procedures. Protein, including urine protein, was determined by BCA Protein Assay Kit (Pierce, Rockford, IL, USA), using bovine serum albumin as standard. Cytokine array, including IL-6, MCP-1, TNF-*α*, leptin, IL-1*β*, and IL-10, was designed for simultaneous detection of these parameters of inflammation by ELISA (Signosis Inc., Santa Clara, CA). Measurements are expressed as % control and were detected using standard methods for ELISA [1]. QRT-PCR was performed to determine the mRNA expression of IL-6, MCP-1, and TNF-*α* in adipose, muscle, and hepatic tissue, using standard methods for PCR [11], in which cycle thresholds (C_t_) for the expression of the gene of interest are compared to C_t_ for *β*-actin. Primers were

 IL-6 (NM_031168):
 (left) 5′-acttccatccagttgccttct-3′ and
 (right) 5′-tttccacgatttcccagaga-3′,
 MCP-1 (NM_011333.3):
 (left) 5′-atctgccctaaggtcttcagc-3′ and
 (right) 5′-taaggcatcacagtccgagtc-3′,
 TNF-*α*: (NM_013693.3):
 (left) 5′-cccctttactctgaccccttt-3′ and
 (right) 5′-ctgtcccagcatcttgtgttt-3′.

### 2.9. Statistics

Results were analyzed using InStat3 software (GraphPad, La Jolla, CA, USA) and are presented as mean value ± standard deviation (S.D.) of at least three independent biological samples with technical replicates of each determination. Standard error of the mean (SEM) was used in the analysis of the rates of appearance and disappearance of glucose in the hyperinsulinemic-euglycemic clamp studies, in which statistical analysis was performed by unpaired *t*-tests, two-sided. In other analyses, unless stated otherwise, the differences among groups were determined by one-way ANOVA with Tukey–Kramer posttest for multiple comparisons. *P* value ≤0.05 is represented by ∗; *P* ≤ 0.01 by ∗∗ and *P* ≤ 0.001 by ∗∗∗. Area under the curve in the glucose tolerance tests was calculated using the formula (*a*2 − *a*1)/2∗(*b*2 − *b*1), where *a* = values of *x*-axis points and *b* = values of *y*-axis points [31].

## 3. Results

### 3.1. Glucose Tolerance Tests following Short-Term Exposure to Carrageenan

**Figure 1:**
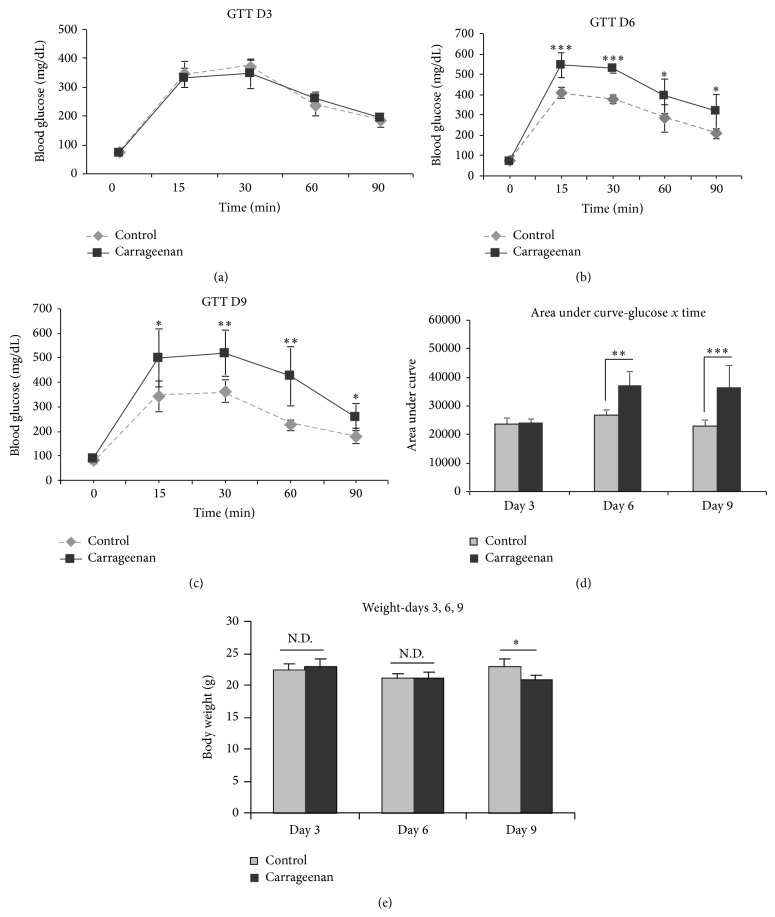
Glucose tolerance tests (GTTs) on days 3, 6, and 9. (a) GTT showed no differences in blood glucose levels between control and carrageenan-exposed groups at 3 days (*n* = 8, 4 per group). (b) At 6 days, the carrageenan-exposed group demonstrated significant elevations of glucose (unpaired *t*-test, two-tailed; *n* = 8). (c) At 9 days, GTT was markedly abnormal (unpaired *t*-test, two-tailed; *n* = 8). (d) Area under the curve measurements confirm differences between control and carrageenan-exposed mice on days 6 and 9. (e) Weights were similar between the carrageenan-exposed and the control mice on days 3 and 6 and slightly less in the carrageenan-exposed mice on day 9.

Glucose tolerance tests (GTT) were performed in control (*n* = 8) and carrageenan-treated mice (*n* = 8) after exposure for short durations, to determine the minimum amount of carrageenan exposure required to cause glucose intolerance. At 3 days (Figure 1(a)), glucose tolerance was similar to the controls; however, by 6 days (Figure 1(b)) and again at 9 days (Figure 1(c)), carrageenan exposure caused significant elevations at 15, 30, 60, and 90 minutes, compared to control. Area under the curve confirmed significant differences for days 6 and 9. Average weights were similar on days 3 and 6 and slightly less in the carrageenan-exposed mice on day 9 (*P* < 0.05, unpaired *t*-test, two-tailed). The mice on the high fat diet with carrageenan drank less water than the mice on carrageenan without high fat (1.63 ± 0.04 mL/d versus 2.06 ± 0.02 mL/d; *P* = 0.004).

### 3.2. Hyperinsulinemic, Euglycemic Clamp Studies following Carrageenan Exposure

**Figure 2:**
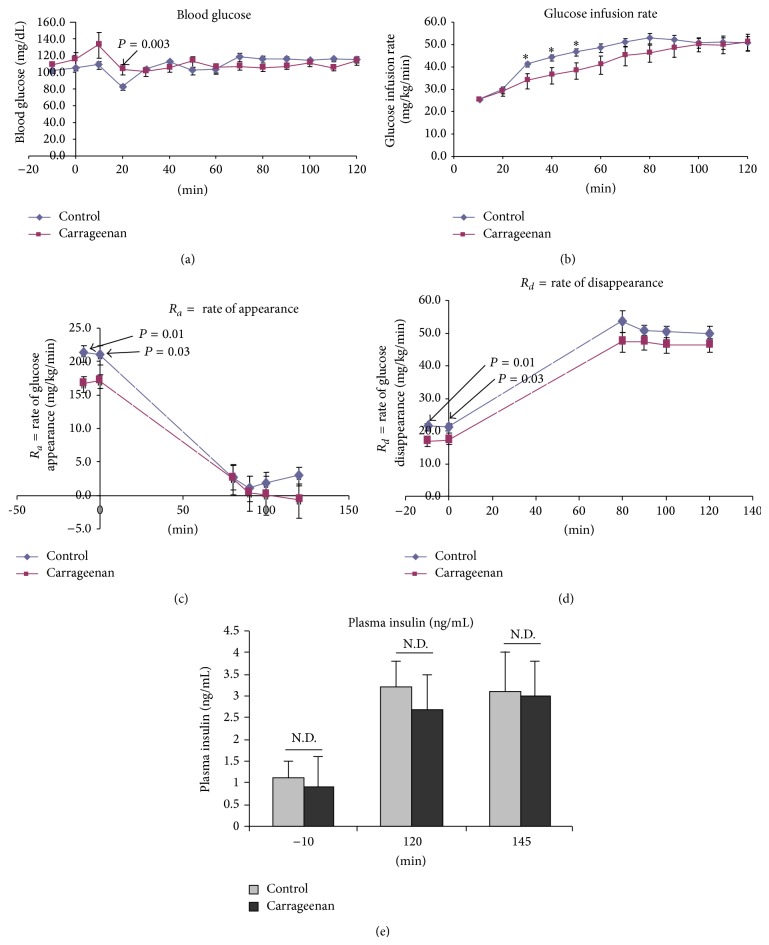
Hyperinsulinemic-euglycemic studies after 18 days of carrageenan exposure. (a) Blood glucose (mg/dL) was higher in the carrageenan-exposed mice than in the control mice at *t* = 20 minutes. Elevation occurred when the glucose infusion rates were similar (see (b)) (*n* per group = 10). (b) Glucose infusion rate (mg/kg/minute) was significantly less in the carrageenan-exposed mice than in the control mice from 30 to 50 minutes, and the interval to achieve steady-state was prolonged by 30 minutes. (c) Endogenous *R*_*a*_ was significantly less at baseline in the carrageenan-exposed mice than in the control mice. At steady-state, values were similar. (d) *R*_*d*_, rate of disappearance of glucose, was significantly less following carrageenan exposure at baseline, but similar at steady-state. (e) Serum insulin levels were similar before and after infusion (N.D. = no difference).

Hyperinsulinemic-euglycemic clamp studies were performed at the Mouse Metabolic Phenotyping Center (MMPC) at Vanderbilt University after carrageenan ingestion for 18 days. At 20 minutes, glucose levels were significantly higher in the carrageenan-exposed mice (Figure 2(a)), although glucose infusion rates were identical (Figure 2(b)). The rise in glucose levels upon initiation of the infusions was steeper in the carrageenan-exposed animals than in the controls. Subsequently, the glucose infusion rate required to maintain euglycemia was less in the carrageenan-exposed mice from *t* = 30 minutes to *t* = 90 minutes (Figure 2(b)). Steady-state was achieved 30 minutes later, from 70 minutes in the control mice to 100 minutes in the carrageenan-exposed mice, consistent with insulin resistance in the nonsteady-state. At steady-state, when the maximal effects of hyperinsulinemia were present, hepatic glucose production was fully suppressed (Figure 2(c)) and glucose disappearance rates were similar in control and carrageenan-treated mice (Figure 2(d)). Plasma insulin levels were similar at baseline and at steady-state between the carrageenan-exposed and control mice (Figure 2(e)). The uptake of ^14^C-2-deoxyglucose, which was infused at 120 minutes, was not significantly greater at any site (Table 2). Overall, findings demonstrate reduced baseline hepatic glucose production, impaired insulin sensitivity with lower rate of glucose infusion to achieve euglycemia, and suppression of endogenous hepatic glucose production in response to hyperinsulinemia in the steady-state.

### 3.3. Long-Term Carrageenan Exposure Induces Fasting Hyperglycemia and Exacerbates Effects of High Fat Diet, without Effect on Weight

**Figure 3:**
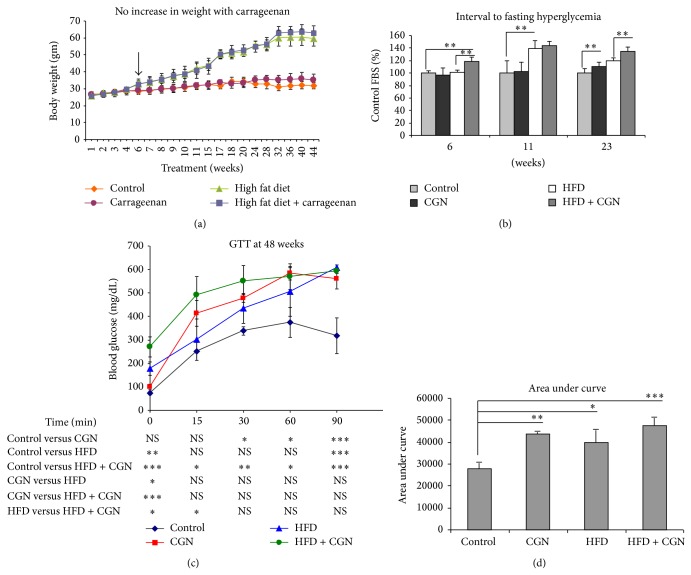
Effects on weight, fasting hyperglycemia, and GTT. (a) Weights were significantly higher in mice on HFD and HFD + carrageenan, compared to control or carrageenan-exposed groups, beginning at 6 weeks and sustained throughout. (b) Fasting blood sugars were significantly higher than control by 6 weeks in HFD + carrageenan, followed by HFD at 11 weeks, and in the carrageenan-exposed group at 23 weeks (*n* = 28). (c) GTT at 48 weeks showed that carrageenan alone produced higher blood glucose levels at 30, 60, and 90 minutes than control. HFD + carrageenan had higher values than control at all time points, whereas HFD value was higher only at baseline (one-way ANOVA with Tukey-Kramer posttest, *n* = 12). (d) Area under the curve confirms significant differences for carrageenan, HFD, and HFD + carrageenan, compared to control. CGN = carrageenan; HFD = high fat diet; GTT = glucose tolerance test.

With either HFD or HFD + carrageenan, the mouse weights increased steadily, compared to the control and carrageenan-exposed mice, and were significantly greater from 6 weeks of the HFD ongoing throughout the study (Figure 3(a); designated by arrow). The mice exposed to the combination of HFD + carrageenan had similar weights to the mice in the HFD alone group.

Fasting blood glucose (FBG) measurements were performed weekly, and in the high fat diet + carrageenan group, significant increase in FBS occurred at 6 weeks. In high fat diet alone, significant increase was present at 11 weeks (Figure 3(b)). In the carrageenan-exposed group, significant differences in FBG compared to control were evident at 23 weeks. Average FBG levels at 23 weeks were 146 ± 10 mg/dL (control), 163 ± 11 mg/dL (carrageenan), 177 ± 7 mg/dL (high fat diet; HFD), and 198 ± 10 mg/dL (HFD + carrageenan).

Glucose tolerance tests performed at 48 weeks showed that carrageenan exposure alone (10 mg/L in water) produced more severe impairment of glucose tolerance than high fat diet (HFD; 58% fat) alone at 15, 30, and 60 minutes (Figure 3(c)). By 90 minutes, the impact of the HFD was similar to that of the carrageenan alone. The combination of HFD + carrageenan produced the highest glucose values in the GTT at baseline and 15 and 30 minutes, indicating that carrageenan exposure increased the propensity of high fat diet to impair glucose tolerance. Area under the curve (AUC) analysis confirmed significant differences in the carrageenan-exposed, HFD, and HFD + carrageenan groups, compared to the controls (Figure 3(d)).

Hemoglobin A1c at 50 weeks was significantly increased in the carrageenan-exposed (6.77 ± 0.49%), the HFD (6.51 ± 0.17%), and HFD + carrageenan groups (6.83 ± 0.34%) (*P* < 0.05), compared to the control group (5.99 ± 0.30%). Spot urine glucose was also increased significantly in the carrageenan-treated mice (*P* < 0.01) and in the HFD + carrageenan group (*P* < 0.05) compared to the controls and HFD alone (data not shown). Spot urine protein was increased following carrageenan, rising to 272 ± 2 mg/dL in the carrageenan alone group and 279 ± 3 mg/dL in HFD + carrageenan, compared to 118 ± 5 mg/dL in the control group and 156 ± 6 mg/dL in the HFD alone group (*P* < 0.001).

### 3.4. Carrageenan-Induced Decline in Hepatic Glycogen by ELISA and by Histochemistry

**Figure 4:**
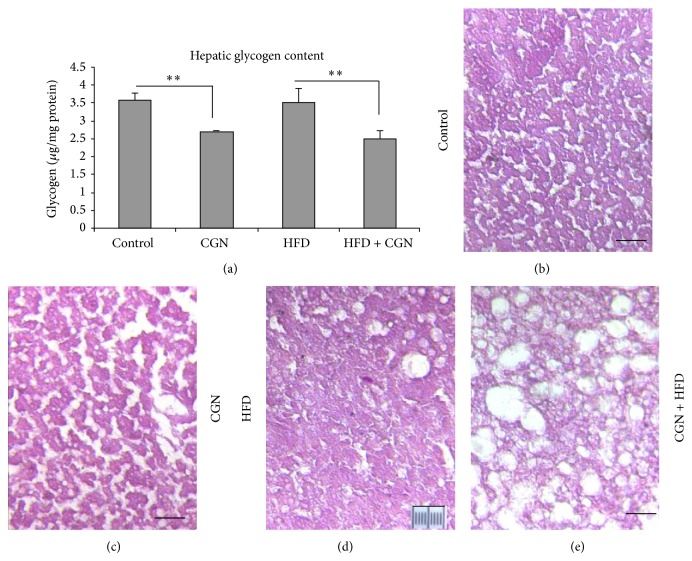
Hepatic glycogen stores declined following carrageenan. (a) Hepatic glycogen was higher in control and HFD groups in blood samples obtained at 50 weeks (*P* < 0.01; *n* = 27). (b)–(e) Representative sections of mouse liver stained for glycogen by periodic acid Schiff (PAS) in control (b), carrageenan-exposed (c), HFD alone (d), and HFD + carrageenan-exposed (e) mice after 52 weeks showed more intense staining in control and HFD tissues. Marker = 10 *μ*m; original magnification 100x; CGN = carrageenan; HFD = high fat diet.

Glycogen differs from carrageenan by the presence of *α*-1,4-galactosidic bonds, in contrast to the *α*-1,3-galactosidic and *β*-1,4-galactosidic bonds of carrageenan. When glycogen was measured in the hepatic tissue from the four groups of mice, values were significantly lower following carrageenan than in control or high fat diet alone groups (*P* < 0.001) (Figure 4(a)). Similarly, when hepatic sections from control, carrageenan-exposed, HFD-exposed, and HFD + carrageenan-exposed mice were stained by periodic acid Schiff reagent, intensity of staining was greater in the control (Figure 4(b)) and HFD (Figure 4(d)) groups than in the CGN (Figure 4(d)) or CGN + HFD (Figure 4(e)) group.

### 3.5. Increased Cholesterol and Lipids following Carrageenan and High Fat Diet

**Figure 5:**
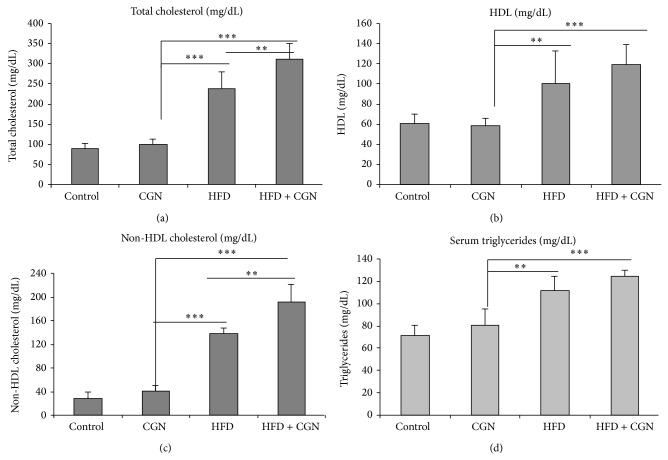
Lipid parameters increased by carrageenan with HFD. (a) Total cholesterol was significantly higher in HFD and HFD + carrageenan mice, compared to control or carrageenan-exposed group (*P* < 0.001, *n* = 27) at 50 weeks, and total cholesterol was more in HFD + carrageenan than HFD alone (*P* < 0.01). (b) HDL cholesterol did not change significantly following carrageenan but was higher in HFD and HFD + carrageenan (*P* < 0.001, *n* = 27). (c) Non-HDL cholesterol did not change following carrageenan but increased following HFD and HFD + carrageenan (*P* < 0.001; *n* = 27) and was significantly higher in the HFD + carrageenan group than HFD alone (*P* < 0.01). (d) Triglycerides increased by HFD and HFD + carrageenan, but not by carrageenan alone (*n* = 27). In HFD + carrageenan, triglycerides were higher than in HFD alone (*P* < 0.01; one-way ANOVA, with Tukey-Kramer posttest, total *n* = 27). CGN = carrageenan; HDL = high density lipoprotein; HFD = high fat diet.

Cholesterol and triglyceride measurements were made after an overnight fast in mice from the four groups after 44 weeks of exposure to carrageenan, HFD, or HFD + carrageenan. The mice on HFD and HFD + carrageenan had significantly higher levels of total cholesterol (Figure 5(a)), HDL (Figure 5(b)), non-HDL cholesterol (Figure 5(c)) and triglycerides (Figure 5(d)) than either the carrageenan-exposed mice or the untreated control (*P* < 0.001; *n* = 28). HFD in combination with carrageenan increased the levels of non-HDL cholesterol and total cholesterol, compared to HFD alone (*P* < 0.01).

### 3.6. Increase in Parameters of Colonic and Systemic Inflammation following Carrageenan

**Figure 6:**
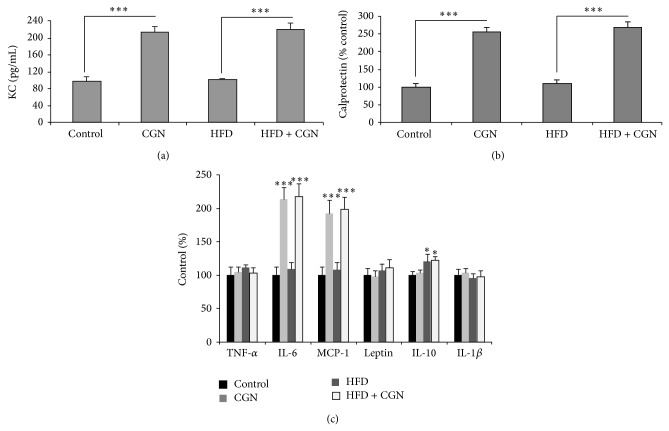
Systemic and colonic measures of inflammation. (a) KC increased following carrageenan, both alone and in combination with HFD after 50 weeks of exposure (*P* < 0.001; *n* = 27). (b) Fecal calprotectin increased in carrageenan alone and in HFD + carrageenan (*P* < 0.001, *n* = 27), but not in HFD alone. (c) Serum IL-6 and MCP-1 were significantly increased following carrageenan and HFD + carrageenan, compared to control and HFD alone in adipose tissue (*P* < 0.001), muscle (*P* < 0.01), and liver (*P* < 0.001; *n* = 3 per group). IL-10 was significantly increased following HFD and HFD + carrageenan (*P* < 0.05; one-way ANOVA with Tukey-Kramer posttest; *n* = 3 per group). HFD = high fat diet; CGN = carrageenan; KC = keratinocyte-derived chemokine.

In the carrageenan-exposed mice (both carrageenan alone and HFD + carrageenan), keratinocyte-derived chemokine (KC), the murine homolog of interleukin-8, was increased from control level of 97.4 ± 10.1 ng/L to 214.6 ± 12.4 ng/L and 220.4 ± 15.6 ng/L, respectively, (*P* < 0.001) at 50 weeks (Figure 6(a)). In the HFD only mice, the KC value was unchanged. Similarly, fecal calprotectin was increased at 50 weeks in the carrageenan alone and HFD + carrageenan groups (*P* < 0.001), but not in the HFD alone group (Figure 6(b)). Measurements of other cytokines demonstrated that IL-6 and MCP-1 were significantly increased following carrageenan and HFD + carrageenan, compared to control and HFD alone (*P* < 0.001, *P* < 0.001) (Figure 6(c)). IL-10 was increased by HFD and HFD + carrageenan, compared to control and carrageenan alone (*P* < 0.05, *P* < 0.05). No differences were evident in serum TNF-*α*, leptin, or IL-1*β*.  Table 1 shows differences in mRNA expression of IL-6, MCP-1, and TNF-*α* in adipose, muscle, and liver tissue following carrageenan, HFD, or HFD + carrageenan by QRT-PCR. Results showed significant increases (*P* < 0.001) in IL-6 and MCP-1 expression following carrageenan and HFD + carrageenan in adipose, muscle, and liver tissue. TNF-*α* expression was significantly increased following HFD and HFD + carrageenan in adipose and liver tissues and in liver tissue following carrageenan alone.

## 4. Discussion

The study findings demonstrate that carrageenan exposure alone led to glucose intolerance after only six days, produced fasting hyperglycemia, reduced glycogen stores, and raised hemoglobin A1c, without increasing weight in the male C57BL/6J mouse. Carrageenan in combination with the high fat diet increased non-HDL cholesterol, accelerated the interval to fasting hyperglycemia, augmented glucose intolerance in response to a glucose load, reduced glycogen stores, and increased systemic and colonic inflammatory parameters. The amount of carrageenan ingested in the mouse studies was less than anticipated in the typical, Western diet. Hence, these results indicate that carrageenan exposure may exacerbate the harmful effects of the high fat diet and contribute to the development of diabetes and atherosclerotic disease in the general population.

The high fat diet is reported to induce glucose intolerance through its effect on inflammation. The current studies showed increases in serum TNF-*α* following the high fat diet, but increases in fecal calprotectin and KC were not detected. However, the high fat mice had increased serum IL-10, suggesting that the increase in IL-10 observed after prolonged feeding might act to inhibit increases in proinflammatory pathways associated with KC and calprotectin. Also, we note the extended interval between onset of abnormal GTT and development of fasting hyperglycemia. We suspect that inhibitory effects of carrageenan on islet function take longer to develop than the rapid effects on the inhibition of insulin signaling. Additional studies are required to clarify the effects on pancreatic function, as well as on the interaction between carrageenan and HFD.

The glycogen assay and histochemical findings demonstrated decline in glycogen stores following exposure to carrageenan either alone or in combination with the high fat diet. These changes are also consistent with the impaired insulin signaling that follows carrageenan exposure and the observed changes in glucose levels in the blood samples. The effects of carrageenan were independent of increase in weight, suggesting that carrageenan may be useful as a nonobese model of diabetes in the mouse.

Carrageenan-induced inflammation appears to be responsible for the glucose intolerance and insulin resistance that follow carrageenan-exposure in the mouse model. The inflammatory properties of carrageenan arise from its activation of reactive oxygen species and from its stimulation of the TLR4 pathway of inflammation [10–12, 32, 33]. The mechanisms shown previously in human hepatic cells and in the mouse hepatic tissue demonstrated that carrageenan-induced inflammation impaired insulin signaling from the insulin receptor, predominantly through increased phospho-Ser307-IRS1, an inhibitor of downstream signaling, leading to reduced phospho-Ser473-AKT [1, 22]. At this time, the mechanism(s) whereby carrageenan exacerbates the impact of the high fat diet is not known, since the inflammatory parameters were not exacerbated by the combined exposures to carrageenan and high fat diet.

Carrageenan structurally closely resembles the endogenous glycosaminoglycans (GAGs) chondroitin sulfate, dermatan sulfate, and keratan sulfate, which contain modified sulfated galactose or N-acetylgalactosamine residues as part of their fundamental, repeating disaccharide structure. This resemblance enables carrageenan to mimic GAG function, leading to inhibition of sulfatase activity and potentially to significant interference with normal GAG metabolism in eukaryotic cells [34–36]. In addition to its inflammation-mediated effects, carrageenan exposure may contribute to the manifestations of diabetes through increases in cholesterol sulfate, arising from its mimicry of GAGs and inhibition of sulfatase enzymes, including steroid sulfatase [35, 36]. Steroid sulfatase removes sulfate groups from cholesterol sulfate, as well as from dehydroepiandrosterone sulfate (DHEA-S), estrone sulfate, and estradiol sulfate [37, 38]. Cholesterol sulfate has been identified in the low density lipoprotein fraction [39, 40]. The presence of cholesterol sulfate in the LDL-cholesterol is consistent with study results that show increased non-HDL cholesterol when carrageenan was given in combination with the high fat diet, compared to the high fat diet alone. Cholesterol sulfate was reported to inhibit sterologenesis and 3-hydroxy-3-methylglutaryl coenzyme A reductase activity in keratinocytes and to increase the incorporation of acetate into fatty acid containing lipids in cultured fibroblasts and keratinocytes in lipoprotein-depleted media, without inhibition of the catabolism of acyl lipids [41].

In our experiments, carrageenan alone had limited impact on the lipid parameters, but the combination of high fat diet and carrageenan significantly increased the non-HDL cholesterol and total cholesterol. Since increased LDL, a component of non-HDL cholesterol, is identified as a risk factor for cardiovascular disease, these study results are consistent with a potential role for carrageenan and cholesterol sulfate in the pathophysiology of atherosclerotic disease. Increased attention to the impact of cholesterol sulfate and to the elimination of dietary ingestion of carrageenan may help in efforts to reduce the incidence of diabetes and its associated morbidities.

## Figures and Tables

**Table 1:** mRNA expression of IL-6, MCP-1, and TNF-*α* following exposure to carrageenan, high fat diet, and the combination of HFD + carrageenan.

Tissue	Adipose			Muscle			Liver		
Fold-change (±S.D.)	CGN	HFD	HFD + CGN	CGN	HFD	HFD + CGN	CGN	HFD	HFD + CGN
IL-6	1.73^***^ (0.15)	1.09 (0.087)	1.78^***^ (0.16)	1.52^**^ (0.11)	0.92 (0.06)	1.50^**^ (0.20)	2.81^***^ (0.23)	1.10 (0.084)	2.84^***^ (0.21)
MCP-1	2.07^***^ (0.018)	1.02 (0.099)	2.01^***^ (0.21)	1.49^**^ (0.11)	1.18 (0.12)	1.52^**^ (0.15)	2.29^***^ (0.17)	1.36 (0.24)	2.67^***^ (0.15)
TNF-*α*	1.07 (0.085)	1.76^***^ (0.10)	1.82^***^ (0.14)	1.06 (0.073)	1.06 (0.12)	1.17 (0.24)	1.50^**^ (0.17)	1.26^*^ (0.055)	1.77^***^ (0.059)

∗: For *P* < 0.05 compared to control; ∗∗: for p<0.01 compared to control, and ∗∗∗: for *P* < 0.001 compared to control.

**Table 2:** Rate of ^14^C-deoxyglucose uptake (*μ*mol/100 g tissue/min) in steady-state (hyperinsulinemic-euglycemic clamp).

Site	Control (*μ*mol/100 g tissue/min) ±S.D. *n*-value	Carrageenan (*μ*mol/100 g tissue/min) ±S.D. *n*-value
Soleus	51.0 (19.8) *n* = 10	67.5 (30.8) *n* = 7
Gastrocnemius	13.3 (4.5) *n* = 11	15.6 (4.1) *n* = 9
Vastus lateralis	13.2 (4.2) *n* = 11	14.4 (4.6) *n* = 9
Adipose tissue	4.8 (2.3) *n* = 11	5.7 (2.1) *n* = 9
Diaphragm	115.4 (38.5) *n* = 11	122.9 (27.2) *n* = 8
Heart	301.8 (73.3) *n* = 11	323.1 (91.0) *n* = 9
Brain	46.3 (10.0) *n* = 11	45.6 (5.7) *n* = 9

## Abbreviations

BCL10: B-cell CLL/lymphoma
CGN: Carrageenan
FBS: Fasting blood sugar
GAG: Glycosaminoglycan
GTT: Glucose tolerance test
HDL: High density lipoprotein
HFD: High fat diet
IRS: Insulin receptor substrate
KC: Keratinocyte-derived chemokine
MMPC: Mouse metabolic phenotyping center
PAS: Periodic acid Schiff
TLR4: Toll-like receptor 4
ROS: Reactive oxygen species
NF-kappaB: Nuclear factor kappa B.
